# The Interplay between the Renin Angiotensin System and Pacing Postconditioning Induced Cardiac Protection

**DOI:** 10.1371/journal.pone.0165777

**Published:** 2016-11-04

**Authors:** Fawzi Babiker, Aishah Al-Jarallah, Shaji Joseph

**Affiliations:** 1 Departments of Physiology, Faculty of Medicine, Health Science Center, Kuwait University, Jabriya, Kuwait; 2 Department of Biochemistry, Faculty of Medicine, Health Science Center, Kuwait University, Jabriya, Kuwait; Virginia Commonwealth University, UNITED STATES

## Abstract

**Background:**

Accumulating evidence suggests a cardioprotective role of pacing postconditioning (PPC) maneuvers in animal models and more recently in humans. The procedure however remains to be optimized and its interaction with physiological systems remains to be further explored. The renin angiotensin system (RAS) plays a dual role in ischemia/reperfusion (I/R) injury. The interaction between RAS and PPC induced cardiac protection is however not clearly understood. We have recently demonstrated that angiotensin (1–7) via Mas receptor played a significant role in PPC mediated cardiac protection against I/R injury.

**Objective:**

The objective of this study was to investigate the role of angiotensin converting enzyme (ACE)—chymase—angiotensin II (Ang II)—angiotensin receptor 1 (AT1) axes of RAS in PPC mediated cardiac protection.

**Methods:**

Isolated rat hearts were subjected to I/R (control) or PPC in the presence or absence of Ang II, chymostatin (inhibitor of locally produced Ang II), ACE blocker (captopril) or AT1 antagonist (irbesartan). Hemodynamics data was computed digitally and infarct size was determined histologically using TTC staining and biochemically by measuring creatine kinase (CK) and lactate dehydrogenase levels.

**Results:**

Cardiac hemodynamics were significantly (P<0.001) improved and infarct size and cardiac enzymes were significantly (P<0.001) reduced in hearts subjected to PPC relative to hearts subjected to I/R injury. Exogenous administration of Ang II did not affect I/R injury or PPC mediated protection. Nonetheless inhibition of endogenously synthesized Ang II protected against I/R induced cardiac damage yet did not block or augment the protective effects of PPC. The administration of AT1 antagonist did not alleviate I/R induced damage. Interestingly it abrogated PPC induced cardiac protection in isolated rat hearts. Finally, PPC induced protection and blockade of locally produced Ang II involved enhanced activation of ERK1/2 and Akt components of the reperfusion injury salvage kinase (RISK) pathway.

**Conclusions:**

This study demonstrate a novel role of endogenously produced Ang II in mediating I/R injury and highlights the significance of AT1 signaling in PPC mediated cardiac protection in isolated rodents hearts ex vivo. The interaction between Ang II-AT1 and PPC appears to involve alterations in the activation state of ERK1/2 and Akt components of the RISK pathway.

## Introduction

Cardiovascular diseases are major health concerns worldwide and coronary heart disease (CHD) continues to be a leading cause of death [1]. Intermittent interruption of coronary flow in the early phases of reperfusion, postconditioning, has proven to be cardioprotective [2]. Recently however well planned postconditioning clinical studies resulted in disappointing negative outcomes [3–6] thus necessitating the requirement of alternative procedures that can successfully be translated to the clinic [7]. Pacing post conditioning (PPC) is a modification of classical ischemic postconditioning that involves changing the stretch pattern of the heart by intermittent dyssynchrony of the left ventricle (LV) contraction at a remote site away from the affected blood vessel [8]. We have previously demonstrated a pronounced reduction in the infarct size and a significant improvement in cardiac hemodynamics in hearts subjected to PPC [9, 10]. Recent introduction of PPC to the clinic demonstrated promising results in ST-segment elevation myocardial infarction (MI) patients [11]. When combined with percutaneous coronary intervention, PPC consisting of 10 episodes of 30 sec right ventricle pacing resulted in a 25% reduction in infarct size [11]. Despite the observed cardioprotection PPC however, resulted in an increased incidence of ventricular fibrillation and paroxysmal atrial fibrillation [11]. This could be due to prolonged pacing as continuous prolonged pacing was proven to be detrimental to the heart [12]. PPC thus appears to be a potentially attractive target. However optimization of PPC maneuver and in depth understanding of its interaction with potentially relevant physiological mediators are challenges that remain to be addressed for an appropriate application of the technique in the clinic.

The renin-angiotensin system (RAS) is a master regulator of cardiovascular physiology and remolding [13]. Key effector peptides of the RAS include angiotensin II (Ang II) and angiotensin (1–7) acting via angiotensin receptors-1 or -2 (AT1 or AT2) and Mas receptors, respectively [14]. While Ang II generally demonstrated to have detrimental effects on the development and progression of cardiovascular disease [15–17], Ang (1–7) was shown to be protective [18]. The exact role of RAS in PPC mediated protection is however not well defined. We have recently demonstrated the involvement of Ang (1–7)-Mas receptor axes in the cardioprotective effects of PPC in isolated rodent hearts [18]. In this study however we were set to determine the role of the other arm of the RAS, specifically Ang II, in PPC mediated cardiac protection. Ang II is a biologically active octapeptide that can be produced systemically by the action of angiotensin converting enzyme (ACE) on circulating angiotensinogen [13] or locally via ACE dependent [19] and independent enzymes including chymase, cathapsin G and Kallikrein [20, 21]. Evidence from clinical studies indicate enhanced chymase activity relative to other Ang II forming enzymes including ACE and cathapsin G in the myocardium 14–21 days post myocardial infarction (MI) suggesting the importance of chymase in post ischemic damage to cardiomyocytes [22]. The role of systemically produced or endogenously synthesized Ang II in PPC mediated protection is however not clearly understood. We hypothesize that exogenously infused and/or locally produced Ang II, acting via AT1, to negatively impact PPC mediated cardiac protection. The objective of this study was therefore to investigate the involvement of ACE-chymase-Ang II-AT1 axes in PPC mediated cardiac protection and map potential downstream signaling pathways.

## Material and Methods

### Materials

All chemicals used in this study were purchased from Sigma Aldrich (Sigma–Aldrich, St. Louis, Missouri) unless otherwise indicated. The following drugs were used in the study and drug doses were selected based on previously published studies: Ang II (100nM) [23], AT1 blocker (irbesartan, 10 μM) [24], chymase inhibitor (chymostatin, 100 nM) [25] and ACE inhibitor (captopril, 100 μM) [26]. All drugs were dissolved in the perfusion buffer.

### Ethics statement

The study was approved by Health Science Center, Kuwait University Ethics Committee for animal use. The study was conducted according to the laboratory’s animal care guidelines at Kuwait University, Kuwait in accordance with the international standards of animal care. Rats were maintained at 22°C on 12-hr light/dark cycle (7 am– 7 pm) and water and food were available ad libitum. Rats were anesthetized with intraperitoneal injection of sodium pentobarbital (60 mg/kg) and heparin (1000 U/kg) was administered through the femoral vein, then were sacrificed by cervical dislocation.

### Animals and Instrumentation

Hearts isolated from male Wistar rats weighing between 270–300 g were used for this study. Heart cannulation and perfusion were performed as described previously [27]. Briefly, isolated hearts were perfused retrogradely with freshly prepared Krebs-Hensleit buffer, pH 7.35 to 7.45 at 37.0 ± 0.5°C and gassed with CO_2_ (5%) and O_2_ (95%). Pacing electrodes were placed on RA appendage to keep the heart beating at physiological rat heart rate. Regional ischemia was induced by occluding left anterior descending (LAD) coronary artery for 30 min. Preload was kept constant at 6 mmHg under basal controlled conditions and perfusion pressure (PP) at 50 mmHg throughout the experimental procedure in all protocols. The perfusion pressure was measured downstream from a branch of the aortic cannula using a “statham pressure transducer” (P23 Db). Constant PP was ensured electronically by means of the perfusion assembly (“Module PPCM type 671 (Hugo Sachs Elektronik- Harvard ApparatusGmbH, Germany”)), an effective system for the accurate adjustment of PP between 5 mmHg to 150 mmHg with ± 1 mmHg accuracy level.

### Study Protocol and Study Groups

**Fig 1:**
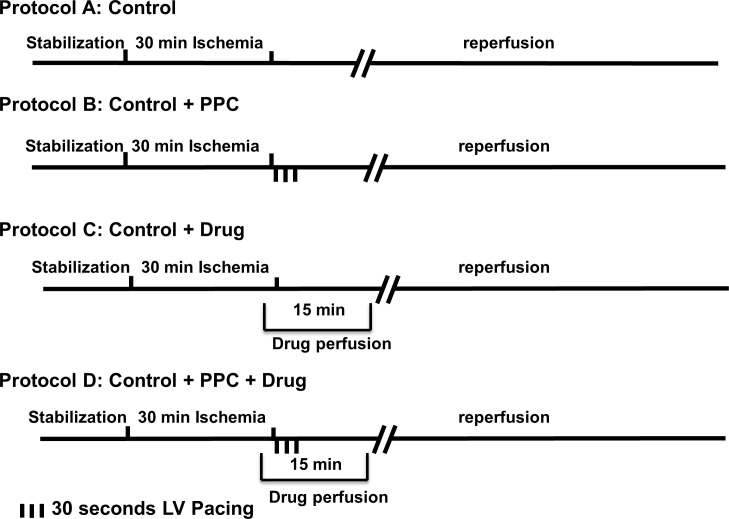
Schematic representation of the experimental protocols used in the study. Hearts isolated from Wister rats were subjected to one of the following protocols (n = 8 hearts per treatment). (A) Unprotected ischemia-reperfusion (control). (B) Ischemia and PPC started immediately at the start of reperfusion. (C) Ischemia and drug dosed 5 min before the start of reperfusion in absence of PPC. (D) Ischemia in presence of PPC and drug dosed 5 min before the start of reperfusion. LV, left ventricle; PPC, pacing postconditioning.

Hearts were subdivided into 10 groups (n = 8 per group). Hearts were subjected to ischemia followed by reperfusion (control) or ischemia followed by LV pacing (PPC) in the presence or absence of target specific drug (Fig 1). Ischemia (30 min) was produced by LAD coronary artery occlusion. The LAD coronary artery was encircled by a snare at approximately 0.5 cm below the atrioventricular groove, and a small rigid plastic tube was positioned between the heart and the snare to ensure complete occlusion of the coronary artery. Hearts were then reperfused for 30 min. In hearts subjected to PPC, one pacing electrode was fixed to the posterior basal LV wall and connected to a pacemaker set to the required pacing frequency. PPC involved three alternating RA and LV pacing episodes, 30 s each. The on and off cycles of pacing were selected based a previous study by our group [10] and were confirmed by a pilot study (data not shown).

### Data Collection and Processing

LV function was evaluated by the assessment of LV end diastolic pressure (LVEDP) and LV developed pressure (DPmax) while coronary-vascular dynamics were evaluated by the assessment of the coronary flow (CF) and coronary vascular resistance (CVR). Cardiovascular functions were measured as previously described [27]. Briefly, a water-filled latex balloon was placed and secured in LV cavity. The balloon was attached to a pressure transducer and a “DC-Bridge amplifier (DC-BA)” with a pressure module (DC-BA type 660, Hugo-Sachs Electronik, Germany) and interfaced to a personal computer for on-line monitoring of DPmax. LV developed pressure was derived from online acquisition of LV end systolic pressure (LVESP) using Max-Min module (Number MMM type 668, Hugo Sachs Elektronik-Harvard ApparatusGmbH, Germany) which has the ability to convert the output from DC bridge amplifier to DPmax by subtracting LVEDP from the LVESP.

Coronary flow was continuously measured using an electromagnetic flow probe attached to the inflow of the aortic cannula interfaced to a personal computer. The continuous monitoring of the CF in ml/min was digitally monitored using software developed by Hugo-Sachs (Hugo-Sachs Electronik, Germany) specifically for this purpose and was manually verified by timed collection of coronary effluent. The CVR and hemodynamics data were determined every 10 sec using an on-line data acquisition program (Isoheart software V 1.524-S, Hugo-Sachs Electronik, Germany). By the end of each experiments, hearts were snap frozen in liquid nitrogen then stored in -80°C for further analysis.

### Assessment of cardiac damage by TTC staining and cardiac enzyme release

Hearts (n = 4 per group) were collected after 30 min of reperfusion and stored overnight at -20°C. The next day the hearts were sliced to 4–5 pieces from apex to base. The slices were then incubated in 1% 2,3,5-Triphenyl-2H-tetrazolium chloride (TTC) solution in isotonic (pH 7.4) phosphate buffer and then fixed in 4% formaldehyde. The red and pale non stained areas of every slice were indicated manually on the image. The percentage of the infarct size of the LV was calculated for every heart. Quantification of the LV and infarct size was done using ImageJ (Image J, Wayne Rasb and National Institute of Health, USA). Cardiomyocytes injury was evaluated by measuring creatine kinase (CK) and lactate dehydrogenase release in the coronary effluent during the reperfusion period as described previously by Ferrera et al. [28].

### Immunoblotting

Left ventricles (n = 4 per group) were homogenized in ice-cold lysis buffer, and the homogenate was centrifuged at 4000 rpm for 10 min. The supernatant was collected, protein content was measured and the samples were aliquoted and stored at -80°C for further analysis. The expression of proteins in question was done as described previously [29]. Equal loading was checked by stripping and reprobing the membrane with Actin antibodies. The levels of total ERK1/2, p38 MAPK and AKT and phosphorylated ERK1/2 (Thr202/Tyr204), phosphorylated p38 MAPK (Thr180/Tyr182) and phosphorylated AKT (Thr308/Ser473) were determined using monoclonal antibodies (Cell Signaling Technology, Inc). Detection was performed with the enhanced chemiluminescence after incubation with a suitable secondary antibody conjugated to horseradish peroxidase (ECL; Cell Signaling Technology). Densitometric analysis was performed using Quantity One software (Biorad).

### Data Analysis

Data were expressed as means ± standard error of the mean (SEM). Hemodynamics data were analyzed with One-way analysis of variance (ANOVA) to determine differences between multiple groups. If an analysis of variance showed a significant difference, post hoc analysis with Tukey test was used for further comparisons. P<0.05 was considered statistically significant.

## Results

**Fig 2:**
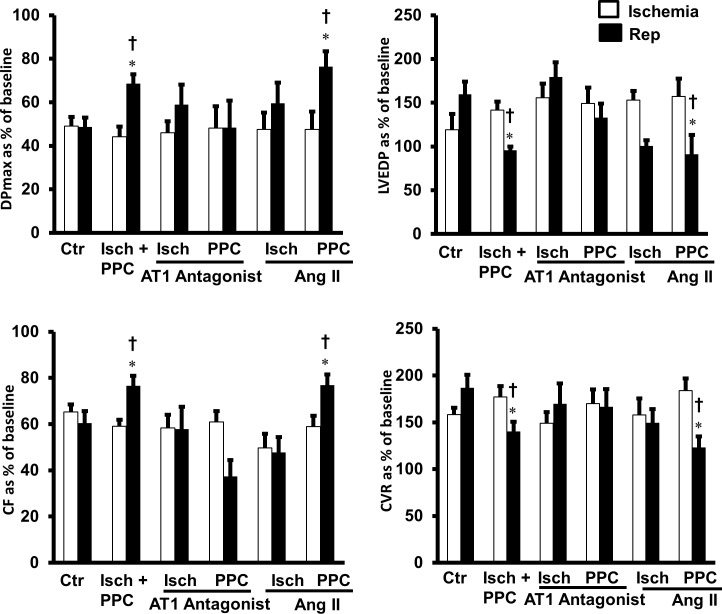
The role of exogenously administered Ang II and Ang II receptor, AT1, in PPC. Isolated rat heart (n = 8 per group) were subjected to I/R injury with or without PPC. Hearts were then infused with Ang II (100nM) or AT1 antagonist (irbesartan, 10μM) and post-ischemic recovery of cardiac functions were recorded. (A) DPmax, (B): LVEDP, (C) CF and (D) CVR. The data were computed at 30 min reperfusion and are expressed as means ± SEM. DPmax, maximum developed pressure; LVEDP, left ventricular end diastolic pressure; CF, coronary flow; CVR, coronary vascular resistance; Ctr, control; Isch, ischemia; PPC, pacing postconditioning. *P<0.05 compared to respective control. ^†^P<0.05 compared to ischemic period.

**Fig 3:**
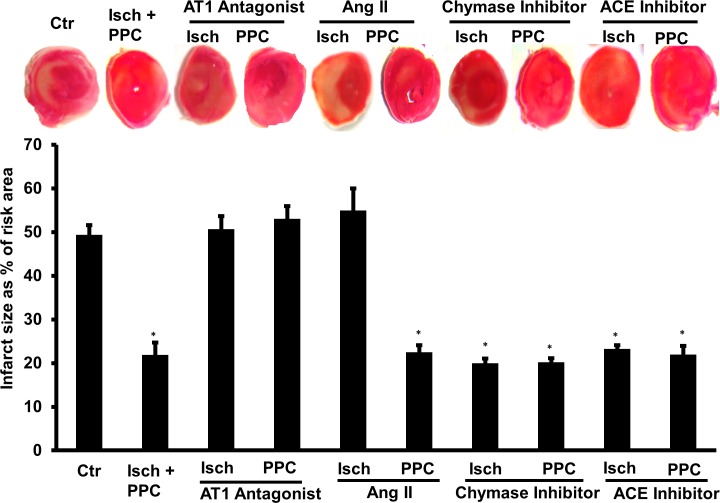
Infarct size determination by TTC staining. Isolated rat hearts at the end of reperfusion (n = 4) were sectioned and stained with TTC (upper panel). Infarct size was quantified and expressed as a percentage of total LV area (lower panel). Ctr, control; Isch, ischemia; PPC, pacing postconditioning.*P<0.001 compared to respective controls.

The role of exogenously administered and endogenously produced Ang II in I/R injury and PPC was examined using recombinant Ang II and ACE and chymase enzyme specific inhibitors, respectively. Exogenously administered Ang II did not affect cardiac hemodynamics (Fig 2), infarct size or cardiac enzymes (Fig 3 and Table 1) in hearts subjected to I/R injury or PPC suggesting that exogenous administration of Ang II does not worsen I/R injury nor attenuates PPC mediated protection of isolated rodent hearts.

**Table 1:** Effects of RAS system on the infarct size and heart enzymes levels.

Treatment	Infarct size	P value	CK	P value	LDH	P value
Control	49.39±2.18	-	49.43±1.70	-	33.63±1.23	-
Pacing at LV	21.78±2.81*	0.001	31.57±1.56*	0.01	14.17±1.47*	0.001
Ischemia + AT1A	50.67±2.92	0.3264	45.73±2.74	0.316	31.07±2.28	0.379
PPC + AT1A	53.00±2.96	0.3786	40.63±3.89	0.107	31.27±2.51	0.445
Ischemia + ANG II	54.94±5.05	0.3658	45.63±0.98	0.126	37.33±1.65	0.147
PPC + ANG II	22.51±1.55*	0.001	19.17±3.12*	0.001	21.00±1.65*	0.01
Ischemia + Ch	20.00±1.01*	0.001	23.20±2.91*	0.001	17.00±2.50*	0.01
PPC + Ch	20.21±0.88*	0.001	26.70±2.91*	0.01	17.17±2.47*	0.01
Ischemia + Cap	23.27±0.83*	0.001	28.07±2.12*	0.001	15.1 ±1.63*	0.001
PPC + Cap	21.96±1.95*	0.001	25.67±2.56*	0.01	17.20±1.10*	0.001

AT1A, angiotensin receptor 1 antagonist; PPC, pacing postconditioning; ANG II, angiotensin II, Ch, chymostatin; Cap, captopril.

*P<0.05 compared to respective controls.

**Fig 4:**
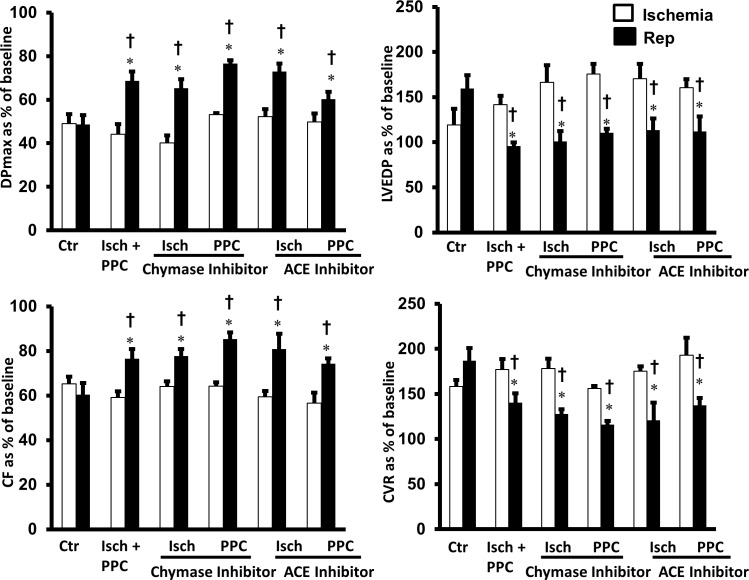
The role endogenously produced Ang II in PPC mediated cardiac protection. Isolated rat hearts (n = 8 per group) were subjected to I/R injury and PPC in the presence or absence of ACE inhibitor (captopril, 100μM) or chymase inhibitor (chymostatin, 100nM). Post-ischemic recovery of cardiac functions were recorded. (A) DPmax, (B) LVEDP, (C) CF and (D) CVR. The data were computed at 30 min reperfusion and are expressed as means ± SEM. DPmax, maximum developed pressure; LVEDP, left ventricular end diastolic pressure; CF, coronary flow; CVR, coronary vascular resistance; Ctr, control; Isch, ischemia; PPC, pacing postconditioning.*P<0.05 compared to respective control, ^†^P<0.05 compared to ischemic period.

In the heart Ang II can be produced locally by ACE-dependent and independent pathways. We therefore tested the involvement of locally produced Ang II in I/R injury and in PPC mediated cardioprotection. The involvement of ACE dependent and chymase mediated intracellular production of Ang II were tested by infusion of captopril or chymostatin at reperfusion, respectively. Myocardial infusion of captopril or chymostatin significantly (P<0.05) protected the heart against I/R injury (Fig 4) and reduced infarct size and cardiac enzymes (Fig 3 and Table 1). The protective effects of PPC on heart hemodynamics, infarct size and cardiac enzymes (Fig 3 and Table 1) however were not augmented by the administration of captopril or chymostatin (Fig 4). All together this data suggest the possible involvement of ACE and chymase dependent production of Ang II during I/R induced cardiac injury while the lack of an additive effect of captopril and chymostatin to PPC mediated protection may however suggest the existence of a common downstream effector pathway. Locally produced Ang II however does not seem to play a significant role in PPC induced protection.

Detrimental effects of Ang II in the cardiovascular system were shown to be mediated by AT1. We have therefore tested the involvement of AT1 in the observed effects of locally produced Ang II using Ang II receptor blocker, irbesartan. We anticipated that blockage of AT1 would reduce I/R injury and may potentially enhance PPC mediated cardiac protection. To our surprise however, infusion of AT1 receptor antagonist did not protect the heart against I/R injury and interestingly revoked the protective effects of PPC on coronary vascular dynamics (Fig 2), infarct size and cardiac enzymes (Fig 3 and Table 1).

**Fig 5:**
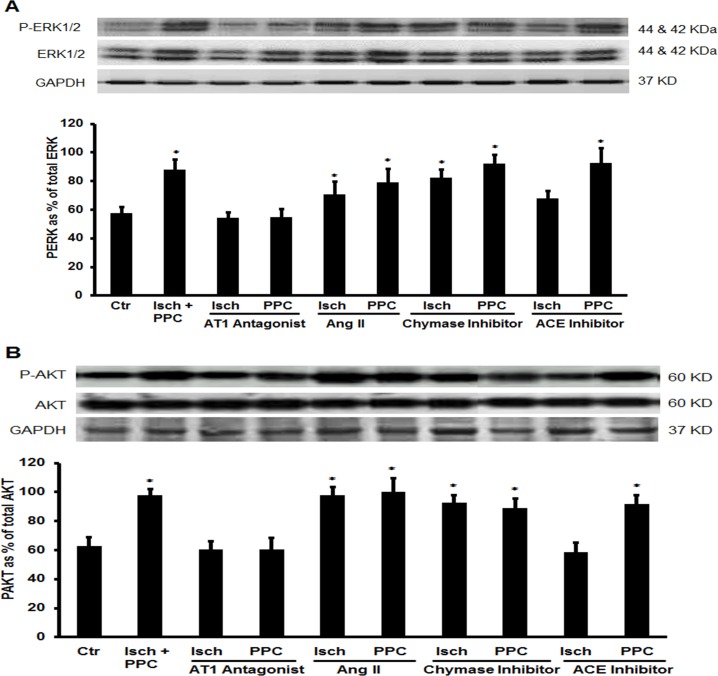
The effect of ACE-Chymase-Ang II-AT1 signaling and PPC on the RISK pathway. (A) Immunoblotting of P-AKT and (B) P-ERK1/2 expressed relative to total AKT and EKR1/2, respectively. Data is presented as mean ± SEM. Ctr, control; PPC, pacing postconditioning. *P<0.05 compared to the untreated control.

To investigate possible downstream signaling pathways involved in PPC mediated cardiac protection we tested the effect of Ang II, ACE inhibitor, chymase inhibitor or AT1 antagonist in presence or absence PPC on the activation state of signaling mediators of the reperfusion injury salvage kinase (RISK) pathway. Ang II treatment did not affect the basal ratio of phosphorylated to total extracellular regulated kinases 1/2 (ERK1/2) or protein kinase B/Akt during I/R injury neither it blocked enhanced phosphorylation of ERK1/2 and Akt in response to PPC (Fig 5). Captopril and chymostatin however significantly (P<0.05) increased ERK1/2 and Akt phosphorylation during I/R injury, yet did not augment PPC-induced phosphorylation of ERK1/2 and Akt possibly suggesting RISK downstream of a common signaling pathway activated by PPC and by the inhibition of ACE and chymase (Fig 5). Blockade of AT1 did not induce ERK1/2 and Akt phosphorylation during I/R injury, however it completely blocked their activation during PPC (Fig 5). This observation is consistent with the lack of an effect of AT1 blockade on cardiac hemodynamics, infarct size and cardiac enzymes levels during I/R injury and elimination of PPC mediated protection. Finally no changes were observed in the phosphorylation state of p38 MAPK during PPC in the presence or absence of Ang II, captopril, chymostatin and irbesartan (data not shown) suggesting that p38 MAPK is dispensable in PPC mediated protection and in PPC interaction with RAS.

## Discussion

**Fig 6:**
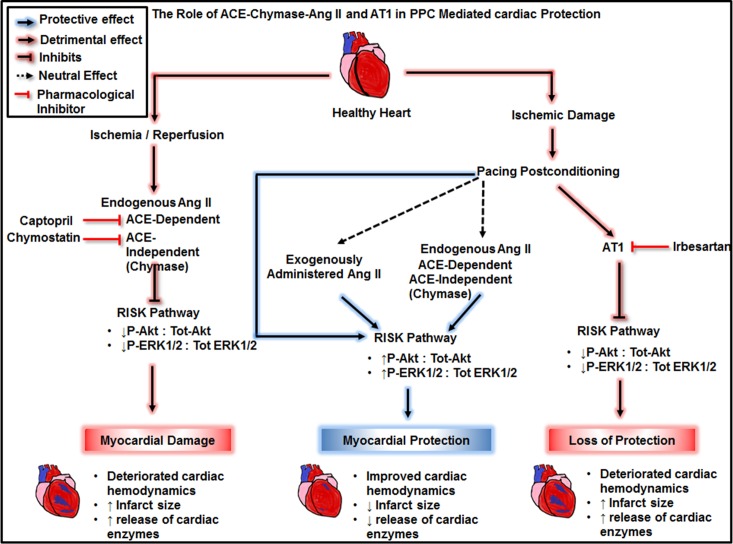
Proposed mechanism of the interaction between ACE-Chymase-Ang II-AT1, I/R injury and PPC mediated cardiac protection. Ischemia/reperfusion induced cardiac injury characterized by impaired cardiac hemodynamics, increased infarct size and enhanced release of cardiac enzymes, LDH and CK, involved ACE and chymase mediated production of Ang II and reduced activation of ERK1/2 and AKT components of the RISK pathway. PPC on the other hand improved cardiac hemodynamic and attenuated infarct size and release of cardiac enzymes. PPC protection involved downstream signaling via the RISK pathway. Exogenous administration of Ang II or blockage of endogenous Ang II had a neutral effect on PPC mediated cardiac protection yet induced the RISK pathway. AT1 antagonist, irbesartan on the other hand abrogated the protective effects of PPC and attenuated the RISK pathway.

In this study we tested the interaction between ACE-Chymase-Ang II-AT1 and PPC mediated cardiac protection in isolated rodent hearts. The main findings of this study are: (1) systemically infused Ang II does not enhance I/R injury nor abrogate the protective effects of PPC. (2) inhibition of ACE or chymase protects against I/R injury yet does not block or augment PPC mediated protection. (3) blockade of AT1 does not protect against I/R injury yet evokes the protective effects of PPC on coronary hemodynamics, infarct size and cardiac enzymes. (4) the interaction between ACE-chymase-Ang II-AT1 and PPC involves signaling via ERK1/2 and Akt members of the RISK pathway (Fig 6).

The RAS is a key regulatory system of cardiovascular physiology in which Ang II is a major effector peptide [13]. Ang II can be produced systemically via ACE dependent pathway or locally via ACE-dependent or independent enzymes [20, 21]. The exact role of RAS in I/R induced injury and PPC mediated ex vivo cardioprotection is however not clearly understood. Local production of Ang II by ACE and chymase is stimulated by stress and ischemic insult [22] and has been implicated in post ischemic cardiac remodeling [30]. Increasing body of evidence indicates the presence of ACE and supports its role in the intracellular production of Ang II in cardiomyocytes [31, 32], heart failure [33] and myocardial infarction [34]. Enhanced chymase mediated intracellular production of Ang II was reported in the vasculature, isolated cardiomyocytes [35], atherosclerotic lesions [36] and infarcted myocardium [22]. The role of ACE or chymase stimulated production of Ang II in postconditioning remains however to be characterized. In this study we demonstrate that blockade of ACE or chymase mediated endogenous production of Ang II, by captopril and chymostatin respectively, alleviates I/R injury. Captopril mediated protection could be due to reduced production of endogenous Ang II or reduction in kinins degradation [37, 38]. This finding sheds light on previously undiscovered role of ACE-dependent and ACE-independent (chymase mediated) endogenous production of Ang II in I/R injury and highlights the existence of potentially, overlooked, targets in the clinical application of postconditioning. Administration of chymase inhibitors to animal models of atherosclerosis, hypertension and heart failure demonstrated multiple beneficial effects including reduced atherosclerotic plaque development [39], improved LV contractility [40] and attenuated post-MI cardiac remodeling with overall improved survival [4]. Furthermore oral administration of chymase inhibitor reduced cardiac injury in a dog model of I/R injury [7]. It cannot be ruled out however that the protective effects of captopril and chymostatin could due to their indirect effects on other peptides besides Ang II [41, 42]. This possibility however remains to be further investigated.

In the cardiovascular system many of the physiological and pathophysiological effects of Ang II are mediated by AT1 receptors [30]. We therefore tested the role of AT1 in PPC mediated cardiac protection. Based on our data from captopril or chymostatin treated hearts, we anticipated that inhibition of AT1 will improve the protective effects of PPC. To our surprise however, PPC mediated protection on cardiac hemodynamic and infarct size were lost upon the infusion of AT1 antagonist (Fig 6, Table 1). This may suggest the possible requirement of AT1 signaling in PPC mediated protection of rodent hearts ex vivo. Signaling via AT1 involved G-protein dependent and G-protein independent pathways [22]. While G-protein signaling has been linked to I/R induced damage, G-protein independent signaling was shown to induce cardioprotection [43]. Simultaneous blockade of G-protein coupled signaling and activation of G-protein independent signaling through AT1 receptor protected against I/R induced injury as indicated by reduced infarct size [43]. Another possible explanation to the loss of PPC mediated cardiac protection in the presence of AT1 antagonist could be due to mechanical stretch induced changes in AT1 signaling and/or localization. Mechanical stretch was reported to activate AT1 receptor independent of Ang II [44]. In addition AT1 was reported to undergo a conformational switch in response to mechanical stretch resulting in inverse agonist-induced inactivation [45]. Tension induced thinning of the membrane lipid bilayer has been suggested as a possible mechanism. However the precise mechanism by which mechanical force is transmitted to AT1 remains to be identified [45]. Similar inverse behavior was shown in α2A-adrenergic receptor whereby inverse agonists resulted in a conformational rearrangements in the receptor and subsequent suppression in activity [46]. Thus its plausible to speculate that pacing induced mechanical stretch may alter AT1 mediated signaling whereby the AT1 antagonist (irbesartan) acts as a receptor agonist. However, this possibility remains to be tested.

The RISK is a cascade of kinase that has classically been implicated in cardioprotection. The pathway involves parallel activation of PI3K/Akt and ERK1/2 upon engagement of g-protein coupled receptors or growth factor receptors resulting in inhibition of glycogen synthase kinase 3β and prevention of subsequent opening of mitochondrial permeability transition pores [47]. Activation of RISK family members ERK1/2 and Akt were shown to confer cardioprotection during reperfusion [48] and postconditioning maneuvers [49–51]. Here we show that PPC induced cardiac protection also involves enhanced activation of ERK1/2 and Akt (Fig 5). In contrast however Pipicz and his group have demonstrated that rapid ventricular pacing does not induce ERK1/2 activation [52]. The reason behind this discrepancy is however not clear. Differences in the pacing procedures used could be a possibility. In addition we demonstrate that the activation state of ERK1/2 and Akt is subjected to regulation by RAS during I/R injury and in PPC-induced protection. Consistent with its effects on cardiac hemodynamics, infarct size and cardiac enzymes levels, exogenously infused Ang II did not change the phosphorylation state of ERK1/2 and Akt during I/R injury and PPC. Inhibition of locally synthesized Ang II by the administration of captopril or chymostatin however significantly induced ERK1/2 and Akt phosphorylation during I/R injury yet did not enhance their activation in hearts subjected to PPC. Suggesting the potential involvement of locally produced Ang II in I/R induced injury and the possible convergence of protection induced by blockage of intracellular synthesis of Ang II and PPC into a common downstream signaling pathway that ultimately stimulates ERK1/2 and Akt. Finally blockade of AT1 did not alter the activation state of ERK1/2 or Akt in response to I/R injury. Inhibition of AT1 however significantly reduced the activation of ERK1/2 and Akt in response to PPC. Likewise this data is consistent with the lack of PPC induced cardiac protection in the presence of irbesartan.

Our study provided insight into ACE-Chymase-Ang II-AT1 role in I/R induced injury and PPC mediated cardiac protection. Findings from this study are likely to have a direct impact on our understanding of the physiological role of RAS in I/R injury and on RAS components that could potentially be attractive pharmacological targets. Our data suggests that AT1 antagonist (irbesartan) offsets the protective effects of PPC. This suggests that hypertensive patients on irbesartan or possibly other AT1 blockers need to be carefully considered in future PPC studies. Henceforth care must be taken when planning studies including this group of patients. Drug interference with postconditioning maneuvers was reported in patients with acute coronary syndrome and was suggested to interfere with postconditioning afforded protection [53], [54]. Our data further suggests that blockade of endogenously synthesized Ang II by captopril or chymostatin post-ischemically might represent a novel pharmacological alternative to PPC for the treatment of myocardial I/R injury as the attained cardioprotection was comparable to that of PPC. The latter finding may have important clinical implications as these drugs are already used in clinical practice and could potentially be safely administered after myocardial infarction. The use of ACE and chymase inhibitors in the treatment of myocardial infarction however needs to be further evaluated at pre-clinical and clinical levels.

## Conclusions

In conclusion this study identifies a previously undiscovered role ACE and chymase induced synthesis of endogenous Ang II in mediating I/R cardiac injury and highlights the significance of ACE and chymase blockage as a potential therapeutic target in the treatment of myocardial infarction. It further characterizes a novel role of AT1 in PPC mediated cardiac protection of rodent hearts ex vivo. Finally it demonstrates that the RISK pathway is a downstream target of PPC and ACE-chymase-Ang II-AT1 signaling.
